# Is problem alcohol use being detected and treated in Irish general practice?

**DOI:** 10.1186/s12875-018-0718-5

**Published:** 2018-02-12

**Authors:** Andrew O’Regan, Walter Cullen, Louise Hickey, David Meagher, Ailish Hannigan

**Affiliations:** 1 Graduate Entry Medical School, Faculty of Education and Health Sciences, University of Limerick, Limerick, Ireland; 2 School of Medicine, University College Dublin, Health Sciences Centre, Belfield, Dublin, Ireland

**Keywords:** Alcoholism, Problem alcohol use, General practice, Primary care, Screening, Brief intervention

## Abstract

**Background:**

The pattern of alcohol consumption in Ireland has serious societal and health consequences. General practice is well placed to screen for problem alcohol use and to carry out brief interventions. The aims of this study were to investigate the prevalence of documentation of problem alcohol use in patient records in Irish general practice, and to describe the documentation of its diagnosis and treatment.

**Methods:**

General practitioners (GPs) affiliated with an Irish medical school were invited to participate in the study. One hundred patients were randomly selected from each participating practice using the practice software and the clinical records were reviewed for evidence of problem alcohol use. The following was recorded: patient demographics, whether problem alcohol use was documented, whether they had an intervention, a psychotropic medication or if a referral was made. Descriptive statistics and an estimate of the prevalence were calculated using SPSS and SAS software.

**Results:**

Seventy one percent of the practices participated (*n* = 40), generating a sample of 3, 845 active patients. Only 57 patients (1.5%, 95% confidence interval 1 to 2%) were identified as having problem alcohol use in the previous two years. 29 (51%) of those with documented problem alcohol use were referred to other specialist services. 28 (49%) received a psychological intervention. 40 (70%) were prescribed psychotropic medications.

**Conclusion:**

This is the first large scale study of patient records in general practice in Ireland looking at documentation of screening and treatment of problem alcohol use. It highlights the current lack of documentation of alcohol problems and the need to re-inforce positive attitudes among GPs in relation to preventive work.

## Background

A recent WHO report showed that almost half of Irish drinkers engage in heavy drinking on a regular basis, placing Ireland’s binge drinking rates at the second highest of 174 countries studied [1]. According to the Central Statistics Office, Dublin, the current per capita alcohol consumption in Ireland is 10.9 l per person aged over fifteen years, a figure that has trebled over four decades, while in most other countries it has fallen [2]. The increase in per capita consumption is associated with an earlier age of commencing drinking [3] and a recent survey showed that half of Irish 15–16 year olds had consumed alcohol and 23% had been intoxicated at least once [4]. Furthermore, a study of third level students showed that two thirds were engaging in hazardous drinking [5]. These figures, particularly, are alarming as people with serious drug and alcohol problems commence drinking at a much earlier age than those without problems [6]. Ireland’s unhealthy relationship with alcohol is therefore related to both the amount and pattern of alcohol consumption.

The socio-economic impact of alcohol in this country is enormous with a cost to the state of 3.7 billion yearly in terms of health, crime and work-place [7]. Alcohol misuse permeates all facets of Irish society and is a factor in: higher-risk sexual behaviour [8], almost half sexual assault cases [9]; in one quarter of marital disharmony and one third of domestic abuse incidents in this country [10]. There is a proven link to suicide [11], with alcohol detected in the blood of a half of suicide deaths in Ireland [12]. From a health perspective, alcohol is a causal factor in more than 60 medical conditions including hypertension, liver cirrhosis and depression [13] and is one of the most important causes of cancer in Ireland. It is a proven factor in the aetiology of cancers of the mouth, throat, stomach, liver and breast [14]. 28% of emergency department admissions to six of Ireland’s main hospitals are due to alcohol with half of these being in the 18–30 year old age-group [15], mostly due to binge drinking.

Successive legislation has been largely ineffective in addressing the alcohol crisis but the seriousness of the problem has been recognised in recent legislation (Public Health [Alcohol] Bill, 2015) which includes evidence-based measures such as minimum unit pricing. Health strategy has emphasised the importance of resources and training for the preventative role of primary care. It is recognised that general practitioners (GPs) commonly see patients with a range of alcohol-related risks and problems (8–18% of patients presenting to primary care) and screening and brief interventions in this setting are proven to reduce misuse levels [16]. GPs have been identified as appropriate professionals to screen for those at risk of problem alcohol use and to conduct brief interventions to influence patients to think more actively about their alcohol consumption [17]. Despite the magnitude of the national alcohol problem and the detrimental effects on health and society, there is a surprising lack of data from general practice on the documentation of alcohol use and treatment.

The aims of this study, therefore, were to investigate the prevalence of documentation of problem alcohol use in patient records in Irish general practice, to describe the documented treatment of patients with problem alcohol use and their psychological co-morbidities.

## Methods

### Participants

This study is part of a larger study on prevalence of psychological morbidity in adults attending general practice in Ireland, the methodology of which has been previously described [18]. All general practices affiliated with the University of Limerick Graduate Entry Medical School with a senior medical student on clinical placement in 2013 (*n* = 56 practices) were invited to participate in the study. As part of the medical school professional competencies curriculum, third year medical students are required to complete a clinical audit or research project and may do this while on any placement. A unique aspect of the curriculum is an 18-week general practice placement where faculty provide a standardised project for students with ethical approval, instructions on data retrieval and analysis, a coding sheet and supervision for the duration of the project. With this level of support and the suitability of electronic records to audit and research, the majority of students opt for the standardised project in general practice in conjunction with their GP tutors.

Practices affiliated with the School are broadly representative of practices nationally by size, urban/rural and patient eligibility for free care. Reporting functions of electronic practice management systems in all participating practices were used to generate a list of all patients aged 18 and over with a documented contact to the practice in the previous two years. A random sample of 100 adult patients was selected from this list in each practice using a random number function in Microsoft Excel. Inactive patients, e.g. temporary visitors or those known to have recently moved away or died were excluded from the sample.

### Measures

Clinical records for a two year time period (2011–2013) were reviewed for the sample of patients by the senior medical student on placement in the practice and their supervising GP for any evidence of attending the practice with problem alcohol and/or substance use other than alcohol. Evidence included text in consultation notes; evidence of a pharmacological treatment or psychological intervention by the GP; evidence of a referral to another primary healthcare professional or specialist agencies and/or diagnostic coding.

Data was collected using an instrument validated for morbidity surveys in primary care in Ireland [19] which recorded the following information on all patients in the sample:

- Demographics: age, gender, eligibility for free care. The Republic of Ireland has the only health system in the European Union which does not offer universal coverage for primary care [20]. It has a mixed private-public system with 43% of the population eligible for a means-tested General Medical Services (GMS) card or doctor visit only card which is issued by the Health Service Executive. To qualify for a medical card the applicant must earn below a certain figure based on family size. GPs (who are self-employed) are paid a ‘per capita’ fee by the state for their care. These patients do not pay directly for GP consultations whereas patients without a card pay an average of €50 per consultation.
- Health service utilisation: total number of visits to the GP for any reason in the past year; any referral to or attendance at secondary care (including the emergency department) in the past year.
- Whether problem alcohol use or other substance use had been documented in the clinical records over the previous two years and if yes,
  - whether a referral to another agency was made;
  - whether psychotropic medication was received;
  - whether a psychological intervention was received and if yes, the type and delivery of the intervention.

Data was entered to an Excel file in each practice and anonymised datasets from all practices were merged together with practice characteristics (urban or rural, number of patients, number of staff). Ethical approval for the study was granted by the University Hospital Limerick Research Ethics Committee.

### Statistical analysis

The proportion of patients with problem alcohol or substance use documented in the previous two years was estimated with a 95% confidence interval for the proportion, accounting for the structure of the dataset with patients clustered within practices. Demographic and healthcare utilisation variables were summarised using graphical and numeric descriptive statistics. For those patients with problem alcohol and/or substance use documented, information on treatment was summarised using graphical and numeric descriptive statistics. The association between categorical variables was tested using chi-square tests and median consultations rates were compared across groups using non-parametric tests. A 5% level of significance was used for all statistical tests. SPSS Statistics Version 21 for Windows and SAS software Version 9.2 for Windows (SAS Institute, Inc.) were used to carry out the analysis with the SAS procedure SURVEYFREQ used to account for clustering.

## Results

Forty (71%) of the 56 practices affiliated with the Medical School agreed to participate. Practice characteristics of those who did not participate were similar to those who participated. Practice size ranged from less than one thousand to over three thousand registered patients. Twenty two (54%) practices indicated they were urban, 13 (33%) indicated they were rural and 5 (13%) indicated they were mixed urban/rural practices. Of the 4000 patient records sampled across 40 practices, 155 (4%) temporary visitors to the practice or those who were known to have died or moved away were excluded giving a sample of 3845 ‘active’ patients.

### Problem alcohol use

Fifty-seven (1.5%, 95% confidence interval 1 to 2%) were identified as having problem alcohol use documented in the previous two years. Of the 40 participating practices, 14 (35%) had no patients in their sample with documented problem alcohol use, 16 (40%) had up to 2% of their sample with documented problem alcohol use and 10 (25%) had between 2 and 8% of their sample with documented problem alcohol use. Patients with problem alcohol use were more likely to be male than those without any problem alcohol use documented (65% vs. 47%, *p* = 0.007). They were also more likely to be eligible for free GP care (72% vs 48%, *p* < 0.001), have been referred or attended secondary care in the past year (79% vs 50%, p < 0.001) and attend the GP more frequently (median of 5 vs 2 consultations a year, *p* = 0.02) (see Table 1).

**Table 1:** Demographics and healthcare utilisation by whether problem alcohol use was documented

	No problem alcohol use documented (*n* = 3788)	Documented problem alcohol use (*n* = 57)	*P*-value
Median Age (25th, 75th percentile)	46 (32, 61)	49 (36, 58)	0.45
Male	1777 (47%)	37 (65%)	0.007
Eligible for free GP care	1828 (48%)	41 (72%)	< 0.001
Any referral/attendance to secondary care in the past year	1910 (50%)	45 (79%)	< 0.001
Median GP consultations (25th, 75th percentile) in the past year	2 (1, 6)	5 (2, 7)	0.02

The majority (72%) of those with problem alcohol use were identified from free text in consultation notes. Six patients (11%) had a diagnostic code entered for problem alcohol use. Four (7%) patients were identified from referral letters, four (7%) from both referral letters and consultation notes, one (2%) from a ‘past history’ page and one (2%) from a psychological assessment. Of the 57 patients with documented problem alcohol use, 22 (39%) also were documented as having depression, 21 (37%) stress and anxiety and 3 (5%) psychosis.

Twenty-three (0.6%, 95% confidence interval 0.4 to 0.9%) were identified as having substance use other than alcohol documented in the previous two years. The majority of practices (*n* = 25 practices, 63%) had no patients in their sample with documented problem substance use. Of the 23 patients with problem substance use, two (9%) were also identified as having problem alcohol use.

Twenty-nine (51%) of those with documented problem alcohol use were referred to other specialist services. 28 (49%) received a psychological intervention, mostly counselling or a brief intervention. The psychological intervention was given in a diverse range of settings including primary care teams (32%), rehabilitation or addiction services (21%), and Alcoholics Anonymous (4%). 40 (70%) were prescribed psychotropic medications during the two-year timeframe of the study, most commonly antidepressants (47%) and benzodiazepenes (37%). 16 (28%) had two or more drugs prescribed for psychological problems.

## Discussion

### Interpretation of findings

The high response rate to the study among general practices (70%; *n* = 40 practices) yielded a large study population (*n* = 3845 active patients) that is reflective of general practice nationally [21]. The proportion of patients with documented problem alcohol use was 1.5% (95% confidence interval 1 to 2%) and the proportion with documented substance use other than alcohol was 0.6%. Only two of the study population had documented co-existing drug and alcohol abuse. The percentage of patients that have documented problem alcohol use is very small with no patients with documented problem alcohol use in the samples from over a third of participating practices. Given how rare the documentation is, our study cannot explain the factors influencing documentation rates. Previous investigation of clinical documentation suggests that practices that have more user friendly software, as well as more clinician motivation and training, have superior documentation [22] but barriers and enablers to clinical documentation in this context requires further research.

Prevention in primary care in Canada, particularly preventive interventions that involve discussion, e.g. smoking cessation, are often not documented by the GP in the clinical notes [23]. In Ireland, coding is not routinely performed in general practice and notes are written in free text. A key motivator for good clinical note taking in Ireland is protection from litigation which is not relevant to the documentation of unstructured and un-resourced preventive screening. In this context, it is worth considering the outcome of a recent study that found no association between the quality of clinical documentation and the quality of care [24].

Factors that were found to be significant among those with documented problem alcohol use compared to those that did not were: male gender and eligibility for free GP care. The rates of co-morbid depression and anxiety were high in those with documented alcohol use (39 and 37% respectively). Approximately half of those with documented problem alcohol use were referred; half were given a psychological intervention and 70% were prescribed psychotropic medications.

### Comparison with the literature

The documentation rate of problem alcohol use is very low considering that one fifth of patients that present routinely to general practice are likely to be excessive drinkers [25]. The figure is in fact consistent with international findings that, in the absence of interventions, problem alcohol use is underdiagnosed [26], with a large-scale American population study finding that only 5 .5% had been asked about their drinking by their GP [27]. Detection rates are similarly low in Irish hospitals with only 17% of attendees at a tertiary emergency department being asked about their alcohol consumption [28].

The high rate of psychiatric co-morbidities, especially depression and anxiety, and psychotropic prescribing among those identified with problem alcohol use is consistent with international findings, and most likely accounts for some of the higher health service utilisation by the this group. A large scale epidemiological study found that 40% of patients with alcohol abuse problems met the criteria for a psychiatric disorder, typically anxiety and depression [29]. Importantly, that study also highlighted that when problem alcohol use is identified and treated, this proportion falls considerably. The subgroup of patients with documented co-existing drug and alcohol use was very small and is most likely explained by lack of documentation rather than true prevalence.

The high intervention and referral rates in this study may be explained by the fact that those who are detected are more likely to be on the severe end of the spectrum and health related issues would have already surfaced. The hidden majority of problem alcohol users who are not documented could be the group that would benefit most from a brief intervention from their GP [30]. Most GPs, however, prefer not to do the counselling of non-dependent problem drinkers themselves, but to refer to a professional trained in behavioural interventions [31]. Anderson showed that the most important factors necessary for GPs to carry out interventions were: education, support and confidence [32].

### Enablers and barriers to screening for problem alcohol use

The gap between actual practice and potential for preventive work has been previously reported, with busyness and lack of doctor confidence cited as the key barriers [33]. Time constraints, however, should no longer restrict a GPs ability to screen as physician-conducted screening can take 74 s and single question screening has been validated for use in primary care [34]. There may be deeper underlying deterrents for GPs in documenting problem alcohol use, including protection of their patients. GPs frequently answer questionnaires for health insurance companies relating to mortgage protection and employment suitability with their patients’ permission. It may pose a dilemma for a GP on whether to include documented ‘high-risk activities’ such as binge drinking or alcohol addiction, so not documenting the finding may be an easier approach. Possibly, the high rates of alcohol abuse and dependence among physicians [35] may be a contributory factor in the reluctance to ask about alcohol problems in the general practice population. What is even more concerning is the societal psyche in relation to the alcohol problem which must also affect general practitioners, moving one commentator to write: *‘We have moved from a cultural attitude to alcohol that was ambivalent, to a culture where public and private drunkenness are apparently acceptable’* [36].

### Limitations

The study is dependent on the completeness of consultation notes and coding and previous research in general practice has emphasised the need to improve coding practices in primary care [37]. Given that GPs do not routinely document screening, the 1.5% of patients identified in the clinical notes with documented problem alcohol use cannot be taken as the true prevalence of those identified by their GP as having a problem with alcohol in practice. Furthermore, our results cannot give data on the epidemiology of problem alcohol use in the community, given that all subjects in this study were recruited from those that had attended their general practice within the previous two years. Practices involved in our study are similar to the national profile by demographic factors but may be more academically or research active than other practices because of their involvement with the medical school. It is possible, therefore that the figures reported in this study are higher than the national rate of documentation.

### Suggestions for future research and policy

Highlighting the value of screening and intervention in general practice should be a public health priority because the GP, by simply asking about alcohol, can positively affect drinking behaviour [38]. Five to ten minutes of advice from a GP can lead to reductions of 25–35% in alcohol consumption that are maintained at six months and one year [17]. Training is perceived by GPs as the most useful enabler to perform alcohol screening, and the doctor-patient relationship is the most important feature for the patients, who believe that the GP has an important role to play [39]. The Irish College of General Practitioner guidelines state that GPs and practice nurses should screen routinely and opportunistically by asking about alcohol use and documenting the outcome [40]. Future research should focus on alternative methods for collecting data on alcohol use in general practice. There is an opportunity for qualitative studies with GPs to explore attitudes, barriers and potential enabling strategies to improve screening, intervention and the documentation of problem alcohol use in an Irish context.

## Conclusion

This is the first large scale study of patient records in general practice in Ireland looking at documentation of screening and treatment of problem alcohol use. It highlights the current lack of documentation of alcohol problems in patient records and the need for training and discussion to improve confidence and re-inforce positive attitudes among GPs in relation to preventive work. GPs are ideally placed to detect and intervene due to frequent contact with problem alcohol use and the relationship they have with their patients. Ireland urgently needs a national conversation about its relationship with alcohol and on clinical and service organisation levels, GPs should be central to this.

## Abbreviation

GP: General practitioner
